# House Dust Mite Precision Allergy Molecular Diagnosis (PAMD@) in the Th2-prone Atopic Dermatitis Endotype

**DOI:** 10.3390/life11121418

**Published:** 2021-12-17

**Authors:** Ruperto González-Pérez, Paloma Poza-Guedes, Fernando Pineda, Miriam Castillo, Inmaculada Sánchez-Machín

**Affiliations:** 1 Allergy Department, Hospital Universitario de Canarias, 38320 Tenerife, Spain; pozagdes@hotmail.com (P.P.-G.); zerupean67@gmail.com (I.S.-M.); 2 Severe Asthma Unit, Hospital Universitario de Canarias, 38320 Tenerife, Spain; 3 Diater Laboratories, 28191 Madrid, Spain; f.pineda@diater.com (F.P.); m.castillo@diater.com (M.C.)

**Keywords:** allergy, atopic dermatitis, allergen, *Dermatophagoides pteronyssinus*, endotype, precision allergy molecular diagnosis

## Abstract

Atopic dermatitis (AD) endotyping might be important for developing personalized diagnostic and therapeutic strategies to the different phenotypes. The current study investigated the IgE molecular profile to *Dermatophagoides pteronyssinus* (*D. pteronyssinus*) in a subset of patients afflicted with varying severity stages of atopic dermatitis in a subtropical region subjected to a high perennial house dust mite (HDM) exposure. We selected patients showing a clinically relevant sensitization to HDM with mild-to-moderate and severe AD according to their basal Severity Scoring Atopic Dermatitis (SCORAD) index. Skin prick test (SPT) with standardized mite extracts, as well as a Precision Allergy Molecular Diagnosis (PAMD@) panel including nine different *D.* *pteronyssinus* allergens and the related protein allergenic characterization, were assessed in all serum samples. A total of 80 European American AD patients with the marked T2 endotype confirmed their eligibility for the study. Major allergens (Der p 23, Der p 2, and Der p 1) were present in more than 86% of all subjects, with mid-tier allergens (Der p 5, Der p 7, and Der p 21) reaching up to 65%. A serodominant role for Der p 11 could not be quantitatively confirmed in the present cohort. The proposed component resolved diagnosis (CRD) panel appeared to be sufficient to obtain a precise *D. pteronyssinus* molecular diagnosis in AD patients subjected to a climate-dependent high-mite allergen exposure. The raised seroprevalence of IgE response to Der p 23 confirmed this constituent as a major *D. pteronyssinus* allergen in severe stages of atopic dermatitis. A clinically driven molecular approach appears to be essential to frame a more precise diagnosis and therapy of this heterogeneous allergic condition.

## 1. Introduction

Atopic dermatitis (AD) is a complex inflammatory skin disorder in which an interaction between the genetic predisposition, the skin barrier disruption, an inappropriate immune response, and an abnormal microbial skin colonization contribute to generate a highly heterogeneous clinical phenotype that can be classified according to IgE level [1,2,3]. In contrast to intrinsic AD, the extrinsic (80%) phenotype is characterized by high total and environmental serum IgE levels, eosinophilia, personal and family atopic background, and greater rate of filaggrin (FLG) mutation [4,5]. Thus, AD is nowadays regarded as a systemic type 2 inflammation-driven disease, encompassing a specific CD4+ T helper type (Th) 2 cell response and the activation of other T-cell lineages, such as Th1, Th17/IL-23, and Th22 [6,7]. In addition, clinical observations and experimental studies indicate that house dust mites (HDM) can play an essential role in AD pathogenesis as specific trigger factors in sensitized and genetically predisposed AD patients [8,9]. Considering the fact that AD comprises different disease phenotypes, detailed endotyping based on specific clinical, ethnic, or demographic patient groups has been proposed as a rational approach for characterization and stratification of AD endotypes [10]. The introduction of allergen molecules has had a major effect on analytic specificity and allergy diagnosis. In this regard, precision allergy molecular diagnosis (PAMD@) discriminates cross-reactivity with other allergens from genuine sensitization [11,12], allowing the comprehensive assessment of the patient’s sIgE binding to a panel of individual allergens [13,14].

To date, 39 HDM *Dermatophagoides* spp. (Pyroglyphidae) allergens have been well documented through specific IgE (sIgE) binding or skin test reactivity, finding visible differences in their seroprevalence across regions [15,16]. The dominant causative allergen in a given community may be locally dissimilar among subjects and yet the intended underlying atopic disease [17,18]. In the era of precision medicine, the identification of these HDM sensitization patterns is an essential, enabling conditions for accurate diagnosis and treatment, especially with allergen-specific immunotherapy (AIT), which has been shown to be effective for specific AD endotypes [19,20,21,22]. Herein, the aim of the present study was focused on a customized PAMD@ model approach to investigate the HDM molecular signature in a cohort of European American individuals afflicted with varying severity stages of the extrinsic AD phenotype subjected to a high environmental HDM exposure [23] influenced by subtropical weather conditions in Tenerife, Spain.

## 2. Results

### 2.1. Demographic Characteristics of Patients

We finally selected 80 ethnically European American subjects from the outpatient allergy office—39 females and 41 males, median age 25.0 (8–62) years of age—who met the inclusion criteria and classified into two groups—including 40 individuals each—according to their basal Severity Scoring Atopic Dermatitis (SCORAD) index, considering SCORAD > 40 as a marker for the severe forms of AD [24]. The majority of subjects (81.35%, 48 out of 59 individuals) had a previous clinician-confirmed diagnosis of AD for more than 8.2 years. More than 90% (72 out 80 subjects) had a former family history of atopy and regarding comorbidities, 71 (88.75%) patients were afflicted with allergic rhinitis and/or asthma, and 8 (10%) had a clinically confirmed food allergy (egg, milk, seafood, and/or tree nuts) (Table 1).

### 2.2. Quantification of IgE Serum Responses to the Crude Extract of D. pteronyssinus, HDM Molecular Profile, and Total IgE

All 80 AD patients confirming their eligibility for the study showed a marked Th2-high endotype, featuring elevated peripheral eosinophil count, total IgE, and aeroallergen-specific IgE levels. Total IgE ranged from 41.00 to 17,420.0 IU/mL, with a median value of 1245.0 IU/mL. The severe AD cohort showed a median total IgE value (1958.0 IU/mL) higher than the mild-to-moderate AD group (532.0 IU/mL). Median blood eosinophils showed a value of 375 eosinophils/μL, with a slightly higher median value (390.0 eosinophils/μL) in the mild-to-moderate AD group with respect to those with the severe AD (360.0 eosinophils/μL) phenotype. All 80 enrolled individuals had a positive serum sIgE (≥0.35 kU/L) against *Dermatophagoides pteronyssinus* (*D. pteronyssinus*) crude extract, with a mean value of 77.99 kU/L, ranging from 7.3 to >100 kU/L. Globally, up to 98.75% of patients (79 out of 80) were sensitized to one or more of the nine investigated molecular HDM allergens (i.e., Der p 1, Der p 2, Der p 5, Der p 7, Der p 10, Der p 11, Der p 20, Der p 21, and/or Der p 23).

Regarding major individual molecular allergens, Der p 23 led the sIgE immunoresponse in 78 out of 80 (97.5%) patients, followed by Der p 2 in 76 of 80 (95.0%) subjects, while 69 (86.25%) individuals were positive for Der p 1. Mid-tier allergens—Der p 5, Der p 7 and Der p 21—showed a seroprevalence of 82.5%, 77.5%, and 65.0% respectively, while minor allergens, such as Der p 10, Der p 11, and Der p 20, were sequentially present in 6.25%, 5.0%, and 15.0% of the serum samples in the present cohort.

In relation to the aggregation of allergens, the repertoire of molecules recognized by IgE was widely pleiomorphic, including 23 distinct profiles in 80 subjects (Table 2). A polyclonal pattern to six specific molecules—Der p 1, Der p 2, Der p 5, Der p 7, Der p 21, and Der p 23—was most frequently (36.6%) identified in AD patients, regardless of their basal SCORAD index.

**Figure 1:**
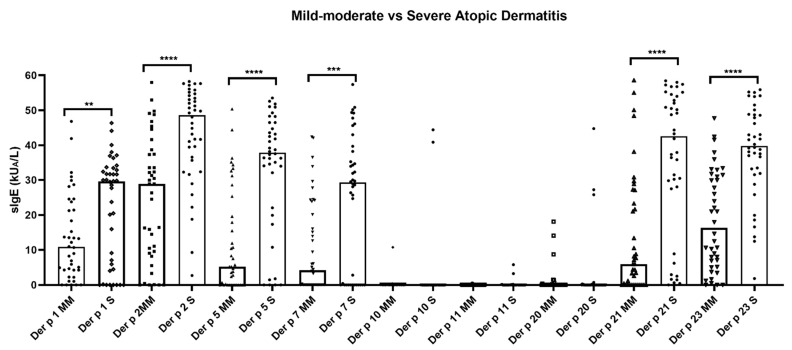
Quantitative average number of *Dermatophagoides pteronyssinus* molecules recognized by IgE antibodies in atopic dermatitis patients (*n* = 80). Median overall values of specific IgE to Der p 1, Der p 2, Der p 5, Der p 7, Der p 21, and Der p 23 showed quantitative significantly (*p* < 0.05) higher titers in the severe atopic dermatitis subjects. No statistical differences were found in the median overall values of specific IgE to Der p 10, Der p 11, and Der p 20 in all patients, regardless of their Severity Scoring Atopic Dermatitis index. Severe: S; mild–moderate: MM; specific IgE: sIgE. ** *p* < 0.01; *** *p* < 0.001; **** *p* < 0.0001.

The quantitative median values of sIgE (kU/L) were significantly (*p* < 0.05) the highest for Der p 2 (48.64 and 28.89) in both groups of AD, followed by Der p 21 (42.56), Der p 23 (39.78), Der p 5 (37.85), and Der p 1 (29.59) in the severe AD individuals and Der p 23 (16.30), Der p 1 (10.8), Der p 21 (5.92), and Der p 5 (5.2) in the mild-to-moderate AD phenotype. In addition, the median overall values of Der p 1, Der p 2, Der p5, Der p 7, Der p 10, and Der p 21 showed statistically significant (*p* < 0.05) higher titers in the severe forms of AD with respect to those patients with the mild-to-moderate condition (Figure 1). Only three patients (3.75%) showed a monomolecular sIgE response to Der p 23 (2.5%) or Der p 2 (1.25%); meanwhile, no subjects were found to be solely sensitized to either Der p 1, Der p 5, Der p 7, Der p 10, Der p 1, Der p 20, or Der p 21.

### 2.3. Sodium Dodecyl Sulphate–Polyacrylamide Gel Electrophoresis (SDS-PAGE) and IgE Western Blot (WB)

**Figure 2:**
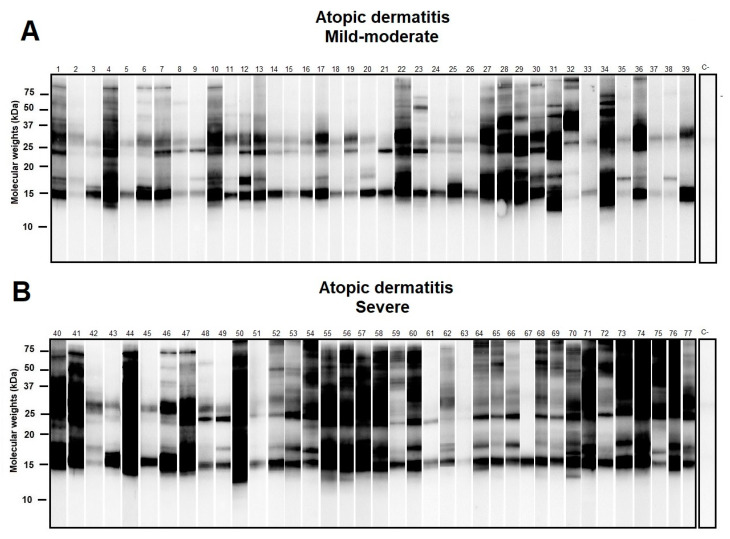
Western blot of atopic dermatitis (AD) subjects (*n* = 80) sensitized to *Dermatophagoides pteronyssinus* (*D. pteronyssinus*) according to their basal Severity Scoring Atopic Dermatitis (SCORAD) index (subfigures (**A**): Mild-Moderate AD and (**B**): Severe AD). A SCORAD index > 40 was regarded as a marker in the severe forms of AD. Although different repertoires of IgE response to *D. pteronyssinus* were found, a notably larger amount of additional protein binding was found in those subjects afflicted with severe AD.

Western blot of selected individuals sensitized to *D. pteronyssinus* showed different repertoires of IgE response with molecular weights ranging from ≈14 to >100 kD. Multiple sensitizations to *D. pteronyssinus* allergens were observed among allergic subjects, especially in those with the severe clinical presentation of AD (Figure 2).

## 3. Discussion

Endotyping AD according to different ethnic backgrounds is essential for establishing disease biomarkers and personalized therapeutic approaches. In this investigation, we used a comprehensive panel of allergen molecules from HDM to characterize the molecular IgE reactivity profile of European American AD patients with a defined clinical T2-driven phenotype. Our findings suggest that patients with severe AD differed from patients with moderate AD regarding several characteristics. In agreement with previous studies [25], severe AD patients showed significantly higher sIgE responses to the proposed HDM component resolved diagnosis (CRD) panel than patients with the mild-to moderate forms of AD. In contrast, despite a more broadly spread sensitization pattern and higher frequency of IgE-reactivity having been previously described [26,27], we found that the same number of HDM molecular allergens—i.e., Der p 1, Der p 2, Der p5, Der p 7, Der p 21, and Der p 23—was recognized across both AD groups, regardless of their severity stage. No differences were found, either in terms of frequency of HDM IgE-immunoresponse, in AD patients, regardless of co-morbid conditions such as allergic rhinitis and/or asthma. The evidence that single allergen components assemble specific additions against a complete mite IgE response suggests that biological functions of particular mite allergens contribute to their allergenicity [11]. In the present study, a high prevailing capacity for sIgE sensitization—based on the frequency of IgE-binding in the population—was found for both HDM major (>86%) and mid-tier allergens (>65%), amongst a well-characterized subtropical cohort with AD under a high perennial HDM exposure. Globally, Der p 23 showed the highest frequency (97.5%) of IgE reactivity in AD patients, closely followed by Der p 2 and Der p1. These findings are coincident with former reports, as the Der p 23-IgE titers of the tested patients were comparable to the IgE levels to Der p 1 and Der p 2 [28]. The elevated prevalence of Der p 23 sIgE further confirmed the serodominance of this peritrophin-like protein, not only in AD but also in related respiratory conditions such as allergic rhinitis and asthma, supporting former investigations from Europe [29,30] and Asia [31]. Although several HDM allergens, -i.e., Der p 5, Der p 7, and the peritrophin-like domain (Der p 21 and Der p 23)- have been quantified as serodominant molecules in the present study, their actual allergenic activity would be only confirmed if the IgE-binding molecules accomplished one positive in vivo and in vitro provocation tests in humans, a positive association with an allergic disease in case–control studies, and a specific proinflammatory pathogenic mechanism [32].

Interestingly, although IgE recognition of allergens mainly present in mite feces (i.e., Der p 1, Der p 2, Der p 5, Der p 7, Der p 21, and Der p 23) was comparable to those described by Banerjee et al. [33], minor allergens such as Der p 10 and especially Der p 11—a paramyosin-like molecule localized in the muscles of HDM and teguments of helminthic parasites—were only present in 1 (1.25%) and 3 (3.75%) out of 80 patients with HDM-associated AD, respectively. The current results are also in agreement with those works published in different European countries showing an overall prevalence sensitization of <4% to Der p 11 in subjects with moderate-to-severe AD [25,34]. Notably, only one out of the three patients with a confirmed double-blind, placebo-controlled food challenge (DBPCFC) seafood allergy diagnosis showed a Der p 11-positive response. These findings may have direct implications in the selection of appropriate extracts for HDM immunotherapy in different populations with variable sIgE HDM profiles and on predicting their therapeutic effectiveness. In this regard, a hypothetical AIT formulation containing Der p 11 may not be of any use in the large majority of the investigated cohort.

The current research has a potential bias as the investigation was performed in only a single center, and the role of minor allergens such as Der p 18, Der f 13, Der f 14, Der f 32, and Der f Alt a 10—regarded as immunologic markers for AD—along with potent proteolytic potential allergens, namely, the serine proteases of the group 3, 6, and 9 allergens, were not tested in the investigated population [35,36,37]. Moreover, one subject (1.25%) with a positive sIgE response to the crude extract could not be identified through our proposed molecular panel, and moreover, other indoor allergens (i.e., storage mites, cats, and/or dogs) might have partially contributed to the severity of the AD. The inclusion of more specific tests, adding further recombinant molecular panels, should be guided by the clinical impact of allergens—an aspect related to IgE-binding but not exclusively to IgE-binding frequency [32]—which may be suitable to overcome this issue [38,39,40].

## 4. Materials and Methods

### 4.1. Subjects

The present investigation was previously evaluated and authorized by the domestic Ethical Committee, and the corresponding informed consent documents were properly signed by all participants—or parents/guardians for those participants <18 y.o.—upon inclusion in the study at Hospital Universitario de Canarias Allergy Outpatient Office and Severe Asthma Unit in Tenerife, Spain, from December 2019 to February 2021. Inclusion criteria required a signed informed consent, a previous clinician diagnosis of present AD, i.e., when atopy was accompanied by chronic relapsing eczematous dermatitis with pruritus [41], and a positive immediate intradermal test to *D. pteronyssinus* (raw extract) with sIgE ≥ 0.35 kU_A_/L to *D. pteronyssinus*. Disease severity and staging was clinically evaluated according to specific guidelines [42]. Severity Scoring Atopic Dermatitis (SCORAD) index formula is A/5 + 7B/2 + C, where A is defined as the extent (0–100), B is defined as the intensity (0–18), and C is defined as the subjective symptoms (0–20), with a maximal score of the SCORAD index of 103 [43]. Pregnant and breast-feeding women and HDM-sensitized patients receiving allergen immunotherapy (AIT), immunosupressors, and/or biologics were excluded from the investigation. Blood specimens were collected, identified with a code label, stored at −40 °C, and immediately thawed in preparation for in vitro analysis.

### 4.2. Skin Prick Test (SPT) and Mite Allergenic Extracts

Percutaneous tests were performed according to European standards [44] with standardized allergenic extracts of *D. pteronyssinus* (Diater, Madrid, Spain). Saline (0.9%) and histamine (10 mg/mL) were included as negative and positive controls, respectively. Antihistamines were dropped back seven days in advance to each SPT, with wheal diameters > 3 mm considered positive after a 20 min immediate reading. Concerning allergenic HDM extracts, proteins from mite bodies of *D. pteronyssinus* were extracted in phosphate-buffered saline buffer 0.01 M, pH 7.4, during 2 h at 5 ± 3 °C. Both protein solutions were clarified by filtration and centrifugation (1 h at 16,000× *g*). The obtained supernatants were then ultrafiltrated to purified water, sterile filtered, frozen, and finally lyophilized.

### 4.3. Serological Workup, SDS-PAGE, and IgE Western Blot

Total IgE levels, sIgE to the whole *D. pteronyssinus* extract, and the 9 individual molecular allergens—Der p 1, Der p 2, Der p 5, Der p 7, Der p 10, Der p 11, Der p 20, Der p 21, and Der p 23—were assessed by ALEX (MacroArray Diagnostics, Vienna, Austria). In brief, ALEX is a multiplex array containing 282 reagents (157 extractive allergens and 125 molecular components). The different allergens and components were coupled onto polystyrene nano-beads, and then the allergen beads were deposited on a nitrocellulose membrane, as previously published [45]. Total IgE levels were expressed in international units per unit volume (IU/mL), and sIgE levels were expressed in kU_A_/L. Values ≥ 0.35 kU_A_/L were regarded as positive. In relation to SDS-PAGE and IgE WB assays, proteins from *D. pteronyssinus* allergenic extracts were analyzed within 15% polyacrylamide gels under reducing conditions, as detailed elsewhere [46]. Proteins were stained by Coomassie Brilliant Blue R-250 and transferred to polyvinylidene difluoride (Trans-blot turbo Biorad^®^, Carlsbad, CA, USA), while IgE-binding was shown by BioRad Diversity database software. The specific binding of IgE to allergens was performed by WB through individual participants’ sera against anti-human IgE peroxidase-conjugate (Southern Biotech, Birmingham, AL, USA), and chemiluminescence reagents (Western lightning^®^, Perkin Elmer, Waltham, MA, USA) were carried out by following the supplier’s indications.

### 4.4. Statistical Data

Demographic features were quoted using average and standard deviation; median, minimum, and maximum values were calculated for the quantitative variables, as well as absolute and relative frequency distribution for the qualitative variables. To quantify deviations in variance, we used both Student’s *t*-test or chi-squared test for parametric continuous, nonparametric continuous, and categorical variables. A *p*-value of less than 0.05 was considered statistically significant.

## 5. Conclusions

In the era of precision medicine, the depiction of the disease-causing allergens by demonstrating the presence of allergen-specific IgE is crucial in AD because it allows for tailoring of different forms of prevention and personalized therapy based on the different clinical and molecular disease subsets. Our findings showed that the currently proposed HDM molecular panel accurately recognized the large majority (>97%) of *D. pteronyssinus*-allergic subjects afflicted with type-2 AD, regardless of their severity stage and the associated comorbidities, in an area with a preponderant load of mite allergen exposure. The tight correlation among IgE responses to Der p 1, Der p 2, Der p 5, Der p 7, Der p 21, and Der p 23, as well as *D. pteronyssinus*, upholds the significance of these allergens in the IgE recognition profile to HDM in our area. The peritrophin-like domain Der p 23 needs to be further investigated as a key component for the diagnosis of AD, with potential therapeutic implications including the use of cost-effectiveness T2-endotype-specific HDM immunotherapy.

## Figures and Tables

**Table 1:** Descriptive statistics of patients with mild-to-severe atopic dermatitis (AD). Severity Scoring Atopic Dermatitis (SCORAD); specific IgE: sIgE; *Dermatophagoides pteronyssinus*: *D. pteronyssinus*.

Descriptive Statistics	Mild–Moderate AD	Severe AD
*n*	40	40
Age (y.o.) median (range)	23.5 (8–53)	26.5 (11–62)
Sex (F/M)	(25/15)	(14/26)
SCORAD median (range)	32 (10–48)	76 (50–96)
Rhinitis and/or asthma *N* (%)	35 (87.5)	36 (90)
Food allergy *N* (%)	3 (7.5)	5 (12.5)
Family history of atopy *N* (%)	37 (92.5)	35 (87.5)
Total IgE (IU/mL) median (range)	532 (41–3158)	1928 (70–17,420)
sIgE D. pteronyssinus (kU/L) median (range)	100 (8.6–100)	55.98 (7.3–100)
Serum eosinophils/mm^3^ median (range)	390 (20–1870)	360 (10–1880)

**Table 2:** Specific IgE profiles to 9 *Dermatophagoides* *pteronyssinus* (*D. pteronyssinus*) molecules examined in 80 subjects afflicted with mild-to-severe atopic dermatitis tested with microarray. Profiles are ordered by the increasing number of recognized *D. pteronyssinus* molecules. Asterisk (*) indicates specific IgE sensitization to single *D. pteronyssinus* molecular allergens.

*n* = 80	%	Number of Molecules	Der p 1	Der p 2	Der p 5	Der p 7	Der p 10	Der p 11	Der p 20	Der p 21	Der p 23
1	1.25	0									
2	2.5	1									*
1	1.25	1		*							
4	5.0	3	*	*							*
1	1.25	3	*				*				*
4	5.0	4	*	*						*	*
3	3.75	4		*	*					*	*
2	2.5	4	*	*	*						*
1	1.25	4		*		*				*	*
5	6.25	5	*	*	*					*	*
4	5.0	5	*	*		*				*	*
1	1.25	5		*	*		*			*	*
3	3.75	5		*	*	*				*	*
2	2.5	5	*	*	*	*					*
29	36.25	6	*	*	*				*	*	*
3	3.75	6	*	*	*	*			*		*
1	1.25	6	*	*	*		*			*	*
1	1.25	6	*	*	*	*				*	*
1	1.25	6	*	*		*			*	*	*
5	6.25	7	*	*	*	*			*	*	*
2	2.5	7	*	*	*	*	*			*	*
3	3.75	8	*	*	*	*		*	*	*	*
1	1.25	8	*	*	*	*	*		*	*	*

## Data Availability

The data that support the findings of this study are available from Servicio Canario de Salud, but restrictions apply to the availability of these data, which were used under license for the current study, and therefore are not publicly available. The data are, however, available from the authors upon reasonable request and with permission of Servicio Canario de Salud.
